# Effect of Breed on the Level of the Nutritional and Health-Promoting Quality of Semimembranosus Muscle in Purebred and Crossbred Bulls

**DOI:** 10.3390/ani10101822

**Published:** 2020-10-06

**Authors:** Paweł Solarczyk, Marcin Gołębiewski, Jan Slósarz, Monika Łukasiewicz, Tomasz Przysucha, Kamila Puppel

**Affiliations:** Institute of Animal Science, Warsaw University of Life Sciences, Ciszewskiego 8, 02-786 Warsaw, Poland; pawel_solarczyk@sggw.edu.pl (P.S.); marcin_golebiewski@sggw.edu.pl (M.G.); jan_slosarz@sggw.edu.pl (J.S.); monika_lukasiewicz@sggw.edu.pl (M.Ł.); tomasz_przysucha@sggw.edu.pl (T.P.)

**Keywords:** breed, meat, quality, crossbreeding

## Abstract

**Simple Summary:**

Beef is an important natural source of nutrients as well as of bioactive ingredients that improve our health. It is an excellent source of alanine, creatine, carnosine, anserine, and polyunsaturated fatty acid (PUFA), which plays an important preventative role in carcinogenic processes. The strategy for rearing and feeding cattle for slaughter should be directed at reducing saturated fatty acid (SFA) in beef fat and/or increasing PUFA, especially n-3. Therefore, the aim of the study was to determine the influence of breed types on the nutritional and pro-health quality of beef. The experiment was conducted in Poland, on 62 bulls from three breeds: Limousin, Polish Holstein-Friesian, and Polish Holstein-Friesian × Limousin. Bulls were slaughtered at 21–23 months of age, and samples of semimembranosus muscle (300 g) were cut parallel to the muscle axis at 24 h postmortem. It can be concluded that commodity crossbreeding significantly improved the quality of beef, resulting in similar or even better results than purebred cattle.

**Abstract:**

Meat from commercial breed cattle are very often used to crossbreed with dairy breeds. The effect of heterosis is most evident when crossbreeds are genetically different from each other. Therefore, the aim of the study was to determine the influence of breed types on the nutritional and pro-health quality of beef. The experiment was conducted on 62 bulls from three breeds: Limousin, Polish Holstein-Friesian, and Polish Holstein-Friesian (PHF) × Limousin. During the fattening period, the animals were fed ad libitum using the same diet. Bulls were slaughtered at 21–23 months of age. The meat of PHF × Limousin hybrids was characterized by the lowest level of SFA and the highest content of n-3 PUFA fatty acids, carnosine, and α-tocopherol compared to the values obtained for the Polish Holstein-Friesian and Limousin breeds. In the case of PHF × Limousin hybrids, there was a 6% increase in n-3 PUFA, 21% in carnosine, and 66% in α-tocopherol compared to the Polish Holstein-Friesian breed. Commodity crossbreeding significantly improved the quality of beef analyzed in this study, resulting in similar or even better results than purebred cattle. This meant that beef from the hybrids with PHF was of the best nutritional and health-promoting quality.

## 1. Introduction

Breed, gender, and the feeding system are factors that determine, to a large extent, the level of bioactive muscle tissue components, because their contribution is closely correlated with the degree of fat cover, and thus is a consequence of diet and genotype [1,2]. Differences between many breeds of cattle have been reported for Red Angus and Simmental steers [3], Aberdeen Angus, Belgian Blue, and Limousine bulls [4], and for different double muscle genotype bulls [1]. Various feeding strategies are often used to increase the content of polyunsaturated fatty acid (PUFA) n-3 fatty acid and to improve beef intramuscular PUFA n-6/PUFA n-3 ratio [5,6,7,8,9]. Beef is an important natural source of alanine, creatine, carnosine, anserine, and polyunsaturated fatty acid, which plays an important preventative role in carcinogenic processes [3,4]. It should be noted that both carnosine and anserine block the production of advanced end-products of glycation, which lead to diseases such as arteriosclerosis, diabetes, cataracts, and Alzheimer’s disease [10,11]. Another important function of these compounds is the formation of complexes with the ions of certain metals, supporting biological activity, which can be exemplified by the inhibition of growth and development of *Helicobacter pylori* [12]. Beef is a source of vitamins soluble in fat. Vitamin A takes part in the processes of growth; it mainly influences the differentiation of the epithelial cells of the oral mucosa, digestive, urinary, and respiratory tracts, and sight organs. Vitamin A catalyzes the oxidation of unsaturated fatty acids, and its presence is essential for the biosynthesis of fat from sugars or from fatty acids and glycerol [13]. The effect of vitamin E is mainly due to its antioxidant properties, thanks to which it protects the body against degenerative diseases [14]. A periodic increase in demand for tocopherols is observed during increased exposure to heavy metals, exposure to free radicals, with a lower supply of other antioxidants, increased physical activity, and in old age [13,15]. Vitamin E prevents the rapid dissipation of ion gradients within muscle reducing rates of tenderization [16,17]. Additionally, Rowe et al. [18] concluded that the use of antioxidants in meat could improve tenderness. Omega-3 and omega-6 fatty acids evoke antibacterial, antiviral, antifungal, antioxidant, and antiparasitic effects [19]. Das [20] reported that the ability of eicosapentaenoic acid (EPA, C20:5 n-3) and docosahexaenoic acid (DHA, C22:6 n-3) to suppress the production of pro-inflammatory cytokines and induce their anti-inflammatory effects results indirectly from their ability to increase mRNA Peroxisome proliferator-activated receptor gamma and protein activity. Additionally, Cheah et al. [21] reported that diets rich in monounsaturated fatty acids (MUFAs) have been shown to have favorable anti-inflammatory and cardiovascular benefits.

Beef is characterized by a moderate and, unlike protein, quite varied fat content, representing 0.7–10% of tissue mass. The composition of intramuscular fat varies from breed to breed, and the proportions between the different types of fiber are different. Late maturing breeds (Belgian Blue, Limousine, and Blonde d’Aquitaine) are characterized by better musculature and lower fat content compared to the parameters achieved by early maturing breeds, e.g., Angus [22]. In addition, Chambaz et al. [23] reported that the Angus breed showed a growth rate similar to Simmental and Charolais while Limousin grew slower, became oldest, and provided the heaviest carcasses and best conformation. The feeding system also influences the fat content of the carcass. The highest content is obtained from intensively fattened animals and the lowest from the extensive fattening method [24,25].

One way to improve low-hereditary functional characteristics is by crossbreeding [26]. This is why there has been a growing interest in this method of animal refinement for several years [27,28]. This method has been recognized as possibly causing heterosis, which is conditioned by the presence of a favorable gene combination. The effect of heterosis is most evident when crossbreeds are genetically different from each other. According to Hansen et al. [28], the effect of heterosis on production traits may amount to as much as 6.5%, while for fertility, health, and survival it may amount to 10%. Meat from commercial breed cattle are very often used to crossbreed with dairy breeds. Carcasses of such hybrids should have a higher proportion of meat and a lower fat content. The beef from these animals is more tender but less marbled than purebred beef cattle. The most commonly used bulls for mating are from breeds such as Limousin, Charolais, Simmental, and Piedmontese, and Hereford, Angus, and Salers are rarely used for this purpose [29]. The offspring of the Limousin and Piedmontese breeds grow rapidly and are well-muscled, and their meat is low in fat and of high quality [30,31].

According to Statistics Poland [32] data, approximately 93% of the cattle population in Poland are dairy cows, and only 1% are cattle of beef breeds. The most popular meat breed in Poland is the Limousin, which is approximately 70% of the population of purebred cows, followed by Charolaise and Hereford. The main criterion for choosing the method of creating a beef herd is economic considerations. Many farmers who decide to create a breeding herd in a short period of time face the problem of high costs associated with the purchase of breeding material. It is possible to reduce such high expenditures by using other methods which, however, involve many years of work. One solution is to create a herd by means of crossbreeding. It is a long-term process, allowing for the gradual elimination of undesired genes and obtaining animals of as similar phenotype and genotype as possible to the paternal breed. Genetic variability in beef quality has been linked to differences between lines or breeds, variations due to the crossing of breeds, and variations between animals [2]. The research hypothesis assumes that commodity crossbreeding Polish Holstein-Friesian (PHF) × Limousin will significantly improve the quality of beef, resulting in similar or even better results than purebred PHF cattle. What is important is that beef production in Poland is mostly related to this dairy breed. Therefore, the aim of the study was to determine the influence of breed (purebred vs. crossbred cattle) on the nutritional and pro-health quality of beef.

## 2. Material and Methods

The experiment was conducted on 62 bulls from three breeds: Limousin (*n* = 25), Polish Holstein-Friesian (PHF, *n* = 12), and Polish Holstein-Friesian × Limousin (*n* = 25). The detailed characteristics of the bulls are presented in Table 1.

The bulls were kept on one farm located at Warmia and Masuria. Cattle were harvested at 21–23 months of age, and hot carcass weight was recorded. Then carcasses were chilled at 2–4 °C and samples of semimembranosus muscle (300 g) were cut parallel to the muscle axis at 24 h postmortem.

Slaughter and postslaughter processing were carried out in accordance with Council Regulation (EC) No. 1099/2009 of 24 September 2009 [33].

The animals were kept in alcoves in a free-standing system, in accordance with the minimum standards of cattle maintenance. During the fattening period, the animals were fed the same diet, ad libitum, with a total mixed ration (TMR) (Table 2).

### 2.1. Chemical Analysis

Beef samples were chopped, then placed in a blender and ground until a homogeneous mass was obtained, which was analyzed later using a near infrared spectrophotometer. The basic chemical composition of meat was determined with a Food Scan™ analyzer (Foss Electric, Hillerød, Denmark).

Meat fat extraction was carried out according to the Folch method [34]. Fatty acid methylation was performed according to the trans-esterification method EN ISO 5509 [35]. Individual fatty acids were identified in crude fat using an Agilent 7890A GC (Agilent, Waldbronn, Germany) with HP Chem Station software (Agilent, Waldbronn, Germany), a flame ionization detector (FID), and a Varian Select FAME column (100 m length, 0.25 mm diameter, 0.25 µm film thickness; Varian/Agilent Technologies, Waldbronn, Germany) according to the methodology by Batorska et al. [36]. The analysis involved a programmed run with temperature ramps under the following conditions and temperatures: the injector was held at 240 °C fitted with siltek deactivated split/splitless liner packed with glass wool (Agilent, Waldbronn, Germany). The column had a total flow rate of 25 cm/s. One microliter of sample was injected with a split ratio of 50:1. The oven method was as follows: 130 °C held for one minute, increased to a temperature of 170 °C at the rate of 6.5 °C/min, then increased to a temperature of 215 °C at the rate of 2.5 °C/min held for 12 min. Then, it was increased to a temperature of 230 °C at the rate of 20 °C/min, held for three minutes. Helium was used as the carrier gas. The FID was operated at 300 °C. All samples were analyzed in duplicate. Each peak was identified using pure methyl ester standards (Supelco, Bellefonte, PA, USA).

The determination of α-tocopherol (vitamin E), α-retinol (vitamin A), and β-carotene were established using an Agilent 1100 Series reverse phase high performance liquid chromatograph (Agilent Technologies, Waldbronn, Germany) according to the methodology by Puppel [37]. Separations were performed at ambient temperature using solvent gradient on a ZORBAX Eclipse XDB column (Agilent Technologies, Waldbronn, Germany). The chromatographic conditions were as follows: Solvent A was MeOH (Merck, Darmstadt, Germany) and water (Sigma-Aldrich, St. Louis, MO, USA) in a ratio of 950:50 (v/v). The flow rate was 1.0 mL/min and the detection wavelength was 280 nm. The injection volume of the final solution was 25 μL. All samples were analyzed in duplicate. The identification of peaks was confirmed by a comparison with the standards (Sigma-Aldrich, St. Louis, MO, USA).

The determination of anserine, carnosine, taurine, coenzyme Q10, creatinine, and creatine was established using Agilent 1100 reversed-phase high performance liquid chromatography (RP-HPLC). Separations were performed at ambient temperature using solvent gradient on the Jupiter column C18 300A (Phenomenex, Torrance, CA, USA) according to the methodology by Łukasiewicz et al. [11]. The chromatographic conditions were as follows: Solvent A was acetonitrile (Merck, Darmstadt, Germany), water (Sigma-Aldrich), and trifluoroacetic acid (Sigma-Aldrich, St. Louis, MO, USA) in a ratio of 30:970:1 (v/v/v). Solvent B was acetonitrile, water, and trifluoroacetic acid in a ratio of 970:30:1 (v/v/v). The flow rate was 1.4 mL/min and the detection wavelength was 214 nm. The injection volume of the final solution was 25 μL. All samples were analyzed in duplicate. The identification of peaks was confirmed by a comparison with the standards (Sigma-Aldrich, St. Louis, MO, USA).

### 2.2. Statistical Analysis

The data obtained were subjected to analysis of variance using ANOVA analysis, with breed as fixed factors. Significant means were separated using Duncan (at *p* < 0.05). The distribution of bioactive components was checked using the Shapiro–Wilk test. All tests were processed using the IBM SPSS 23 package [38]. Data were presented as least squares means (LSM) with standard error of the mean.

The following statistical model was used:

Y*ijk* = μ + A*i* + e*ij* (1)

where: *Yijk* = value of the tested trait; µ = mean; A*i* = effect of the *i* (the breed) (*I* = 1–3); *eij* = standard error.

## 3. Results and Discussion

Many genetic factors influence the quality of beef; the main one is the cattle breed [22,39,40]. They differ in their content and composition of intramuscular fat, the properties of connective tissue, especially collagen, and the proportion between the types of muscle fibers [22]. Table 3 shows a comparison of the protein, fat, and collagen contents in Limousin, Polish Holstein-Friesian, and PHF × Limousin hybrids.

The biological and nutritional value of meat protein is determined by the content of intramuscular connective tissue. Greater protein content in muscle tissue was found in the PHF × Limousin hybrids, at 22.41 g/100 g of meat, and the lowest in Polish Holstein-Friesian at 19.4 g/100 g. In the study by Malczyk et al. [41], the protein content was 21.61 g/100 g in the semitendinosus muscle and 21.51 g/100 g in the longest lumbar muscle in Lowland Black and White × Limousin hybrids. It should be noted that Holsteins may require concentrate-rich instead of silage-rich diets to express their full growth potential [42]. Additionally, purebred Holsteins have a low carcass weight compared to beef breeds or crossbreds [43], which was confirmed by the results obtained in the experiment (Table 1). Studies have shown that the concentration of protein (*p*-value 0.000) was significantly influenced by the breed.

Breeders usually choose animals that are high caliber and late maturing, which achieve very good fattening results. Late maturing breeds such as Limousin, Belgian White, and Blue have less fat and better musculature than early maturing breeds, but less marbling. The Italian and French cattle breeds are characterized by a low proportion of fat and bone and high muscle content. They also achieve satisfactory slaughter yields [30]. European beef cattle, Limousin, and Charolais have, among other features, better slaughter performance, less fat with higher musculature, but less marbling than British breeds such as Hereford or Angus [44]. Table 3 shows that muscle tissue in Limousin cattle was characterized by the lowest level of fat (1.85 g/100 g), and the highest level was found in Polish Holstein-Friesian at 2.95 g/100 g of meat. Studies by Zając [45] showed that the highest fat contents, of 5.30% and 5.15%, respectively, were found in the musculus serratus ventralis and the comb muscle. For comparison, the lowest fat content was in the semimembranosus muscle (2.38%) and semiendodermis muscle (2.77%). In the research conducted by Malczyk et al. [41], the level of fat in Black and White cattle was 2.07% in the heel muscle and 5.03% in the lumbar muscle. Additionally, Nogalski et al. [46] found that Sida silage can improve carcass and meat quality characteristics of Polish Holstein-Friesian bulls. The morphology, composition, and amount of intramuscular connective tissue vary depending on the muscle type, breed, and age of the animal [47].

Another important ingredient in meat is collagen. Table 3 shows that the highest content was found in Polish Holstein-Friesians (592.94 mg/100 g) and the lowest in PHF × Limousins (492.24 mg/100 g). The meat from dairy breeds is characterized by relatively high total collagen content compared with meat obtained from beef breeds [48]. In research by Dąbrowska et al. [49], the following collagen levels were found in meat from Black and White × Limousin crossbred bulls, depending on the muscle tissue analyzed: finger rectifier muscle (musculus extensor digitorum) 3032.2 mg, medium gluteus muscle (musculus gluteus medius) 1506.5 mg, large lateral muscle (musculus vastus lateralis) 878.9 mg, semispongiform muscle (musculus semitendinosus) 525.6 mg, and large lumbar muscle (musculus psoas major) 300.4 mg. Studies have shown that the concentration of collagen was significantly influenced by the breed [50].

The health-promoting quality of meat depends on the saturated fatty acid (SFA) content. A high saturated fatty acid diet has long been implicated with an increased risk of cardiovascular disease [21]. Studies have shown that C12:0, C14:0, and C16:0 have atherogenic properties, while C14:0, C16:0, and C18:0 have thrombogenic properties [22]. Table 4 shows the share of SFAs in Polish Holstein-Friesian, Limousin, and PHF × Limousin hybrids in muscle tissue.

The highest level of SFA, amounting to 49.74 g/100 g, was found in the muscle tissue of Polish Holstein-Friesian cattle. It should be emphasized that this breed exhibited the highest content of all fatty acids studied: lauric acid (C12:0; 0.09 g/100 g), palmitic acid (C16:0; 27.47 g/100 g), and stearic acid (C18:0; 19.26 g/100 g). The levels of the above-mentioned fatty acids in Limousin meat and their hybrids with Polish Holstein-Friesian were similar, especially for C12:0, C14:0, and C16:0. A significant difference was found in C18:0 acid, where in purebred cattle the level of stearic was 18.40 g/100 g, and in hybrids, 16.87 g/100 g. The lowest total content of SFA (43.99 g/100 g) was observed in the PHF × Limousin hybrids. Studies have shown that the concentration of SFA and C18:0 were significantly influenced by the type of breed (*p*-value 0.000). Almeida et al. [51] showed that the formation of a fatty acid profile is also linked to muscle type. Studies have shown that SFA concentration in the biceps femoris is more than three times higher than in the semimembranosus.

Other fatty acids that influence the nutritional and health-promoting quality of beef are monounsaturated fatty acids (MUFAs) and PUFAs.. The main n-3 PUFA in beef are α-linolenic (18:3 n-3), eicosapentaenoic (20:5 n-3), and docosahexaenoic (22:6 n-3) acids [45]. Table 5 presents the content of selected fatty acids in the muscle tissue of Polish Holstein-Friesian, Limousin, and PHF × Limousin hybrids.

Cheah et al. [21] reported that MUFAs may be an ideal substitute for SFA, obviating the adverse effects of SFAs; therefore, the aim should be to increase their share in the diet. The first of the lipid components analyzed was vaccenic acid (C18:1 trans-11; TVA), responsible for the efficiency of tissues and organs. Its highest level was found in PHF × Limousin hybrids (1.31 g/100 g), and its lowest in Polish Holstein-Friesians (0.83 g/100 g).

PUFAs, as essential fatty acids, are only available through dietary consumption [21]. The highest content of LA, 8.70 g/100 g, was found in meat obtained from the Limousins. Slightly lower levels were found in crossbreeds (7.97 g/100 g), and the lowest in Polish Holstein-Friesians (6.24 g/100 g). C18:2 cis-9, trans-11 (CLA) has many functions, which are mainly antioxidant, antiatherosclerotic, and anticancer [52]. The muscle tissue of purebred Limousin and their hybrids with the Polish Holstein-Friesian breed were recorded as 3.26 g/100 g and 3.59 g/100 g, respectively, i.e., more than the 2.59 g/100 g from the Polish Holstein-Friesians. Studies have shown that the concentration of CLA was significantly influenced by the breed.

α-linoleic acid (C18:3 n-3; LNA) plays a key role in hepatic glycolysis, de novo lipogenesis, and fatty acid regulation [53]. The highest level was found in PHF × Limousin hybrids and amounted to 0.71 g/100 g, whereas the lowest was in Polish Holstein-Friesians at 0.49 g/100 g. Other acids analyzed were eicosapentaenoic and docosahexaenoic, which support the functioning of the nervous system. Siscovick et al. [54] reported that the American Heart Association recommends 1 g/day of combined EPA/DHA intake or 2–4 g/day for those with hypertriglyceridemia. Both EPA and DHA were found to be the most abundant in crossbred meat (0.65 g/100 g and 0.16 g/100 g) and the least in Polish Holstein-Friesian meat (0.42 g/100 g and 0.07 g/100 g) (Table 5).

Bioactive dipeptides, including carnosine and anserine, are important components of beef that influence its pro-health value. Table 6 shows the content of bioactive protein fraction components in the muscle tissue of Polish Holstein-Friesians, Limousins, and PHF × Limousin hybrids.

Anserine (β-alanyl-L-(N-methyl) histidine) is a methyl carnosine derivative. It is a dipeptide consisting of β-alanine and L-(N-methyl) histidine. It occurs mainly in skeletal muscles and the brain, and in mammalian organisms it acts as an antioxidant. The concentration of carnosine in raw beef is 300–500 mg/100 g of tissue. For comparison, the average carnosine content of pork is 211–419 mg/100 g tissue. Differences in the concentration of carnosine in muscle tissues result primarily from different levels of muscle oxidation. Carnosine concentration is lower in muscles with high proportions of oxidative muscle fibers [11]. Breed, age, gender, and the rearing system also influence this concentration. The highest level of anserine (83.64 mg/100 g) was found in Limousine meat, and the lowest in Polish Holstein-Friesian meat (61.22 mg/100 g). The carnosine content in crossbreeds was the highest at 492.36 mg/100 g and the lowest in Polish Holstein-Friesians at 387.3 mg/100 g. Mateescu et al. [55] conducted research on the content of bioactive dipeptides in the longest back muscle of the Angus (by sex). The highest carnosine level was found in steers at 370 mg/100 g, in heifers at 367 mg/100 g, and in bulls at 366 mg/100 g. The content of anserine was almost the same in all categories of cattle and ranged from 66 to 67 mg/100 g. Carnosine levels were also determined in muscles and selected organs in a study by Purchas and Busboom [56]. Its content in the longest dorsal muscle was 432.6 mg/100 g, in the semitendinous muscle, 452.6 mg/100 g, and in the musculus triceps brachii, 299.1 mg/100 g.

Taurine, full chemical name 2-aminoethylsulfonic acid, belongs to the amino acids and is commonly found in animal tissues. A lack of taurine in the diet results in a decrease in the number of leukocytes, and in the ability of neutrophils to oxygen burst and phagocytosis. The administration of taurine before an inflammatory reaction or after exposure to oxidative stress prevents or reduces the intensity of pro-inflammatory changes [57]. The highest level of taurine, 48.99 mg/100 g, was found in PHF × Limousin hybrids, and the lowest in Polish Holstein-Friesian meat, 34.28 mg/100 g. Purchas et al. [58] reported significant differences between cheek muscle and semitendinosus muscle.

Coenzyme Q10, also called ubiquinone, is a compound found in every cell of the body and plays a key role in it. Lowering the level of coenzyme Q10 favors the development of diseases arising from, among others, as a result of a reactive oxygen species, e.g., cardiovascular disease or cancer [59]. It is known that as a result of a deficiency of coenzyme Q10, the respiratory chain does not function properly and, as a result, there is insufficient production of high energy compounds [60], which in consequence may reduce the efficiency of the cell, tissue, and the whole organism. It was also found that clinical symptoms associated with deficiency of coenzyme Q10 can be eliminated or reduced by replenishing its quantity in the body with pharmaceutical preparations or dietary supplements [61]. The highest level of coenzyme Q10, 2.67 mg/100 g, was found in PHF × Limousin hybrids, and the lowest in Polish Holstein-Friesian meat, 1.87 mg/100 g. Mattila et al. [62] reported 1.6 mg/100 g for an unspecified beef muscle.

Table 7 shows the content of vitamins soluble in fat in the muscle tissue of Polish Holstein-Friesians, Limousins, and PHF × Limousin hybrids. β-carotene is a precursor of retinol, which is necessary for cell division and differentiation and reproduction [63]. Retinol also participates in the regulation of immune function by supporting the production of white blood cells [64]. Studies have shown that the concentrations of β-carotene and α-retinol were significantly influenced by the types of breed. The study showed almost double the level of β-carotene in the PHF × Limousin group than those in the Polish Holstein-Friesian group. Darwish et al. [65] reported that grass-fed steers had significantly higher amounts (five- to seven-fold) of β-carotene, compared to grain-fed animals, due to the high β-carotene content of fresh grasses as compared to cereal grains.

The highest level of α-tocopherol, 4.76 μg/g, was found in PHF × Limousin hybrids, and the lowest in Polish Holstein-Friesian meat, 1.61 μg/g. Liu et al. [66] reported that a minimum of 3 to 3.5 µg/g meat of α-tocopherol is necessary to prevent discoloration. Additionally, Clausen et al. [67] reported that raw meat with approximately 2 µg/g meat of α-tocopherol showed a high degree of lipid oxidation. Therefore, it can be concluded that increased tissue levels of α-tocopherol protected against retail discoloration and lipid oxidation. It should be emphasized that the quality of muscle tissue varies, with this variability being significantly influenced by type of breed.

## 4. Conclusions

Commodity crossbreeding significantly improved the quality of beef analyzed in this study, resulting in similar or even better results than purebred cattle. The meat of PHF × Limousin hybrids was characterized by the lowest level of SFA and the highest content of omega-3 fatty acids (LNA, EPA, DHA), carnosine, taurine, coenzyme Q10, and vitamins soluble in fat. This meant that beef from the hybrids with PHF was of the best nutritional and health-promoting quality. It should be emphasized that the scheme of crossbreeding Polish Holstein-Friesian or Limousin cattle with other breeds, or in beef herds, should be based on the expectations of the breeder. There are many possibilities from using one or two breeds, or through suppressing crossbreeding. In order for these measures to be profitable, the breeder must consistently follow his breeding strategy. The crossbreeding of beef cattle has two main advantages: hybrid animals show heterosis, and crossbred animals combine the forces of different breeds used to form the cross.

## Figures and Tables

**Table 1:** Bull characteristics on the day of slaughter.

Bull Characteristics	Breed					
	Limousin		Polish Holstein-Friesian		PHF × Limousin	
	Mean	SD	Mean	SD	Mean	SD
Number (n)	25		12		25	
Age (d)	608	12.54	602	11.25	602	11.89
Live weight (kg)	695	35.23	534	24.47	668	34.25
Carcass weight (kg)	412	29.25	317	26.15	382	28.65

**Table 2:** Ingredients and chemical composition of feed (as fed).

Ingredients	Content
Corn silage (%)	67.80
Barley (%)	29.20
Supplement * (%)	3.00
Diet composition:	
Dry matter (%)	54.24
Crude protein (g/kg)	128.40
NEm (Mcal/kg)	1.77
NEg (Mcal/kg)	1.15
NDF (g/kg)	343.23
ADF (g/kg)	193.56
Crude fat (g/kg)	19.20

* The supplement was composed of: 56.5% barley, 10% rape meal, 2% urea, 25% limestone, 3% salt, 0.066% vitamin E 500, 1% premix, 0.05% flavor, and 2.5% molasses, which provided 5% of diet in Dry matter (DM), and supplemented 1 kg diet (in DM) with additional 14.67 mg copper, 58.32 mg zinc, 26.73 mg manganese, 0.66 mg iodine, 0.23 mg cobalt, 0.29 mg selenium, 4.825 IU vitamin A, 478 IU vitamin D, and 32 IU vitamin E.

**Table 3:** The influence of breed on the formation of the basic chemical composition of muscle tissue.

Component	Breed			SEM	*p*-Value
	Limousin	Polish Holstein-Friesian	PHF × Limousin		
Protein (g/100 g)	21.31 ^AB^	19.4 ^AC^	22.41 ^BC^	0.241	0.000
Fat (g/100 g)	1.85 ^A^	2.95 ^AB^	1.89 ^B^	0.035	0.010
Collagen (mg/100 g)	511.68 ^AB^	592.24 ^AC^	492.24 ^BC^	1.231	0.042

A–C: Means in the same row marked with the same letters differ significantly at: capitals, *p* ≤ 0.01. SEM, standard error of least square means (LSM).

**Table 4:** The influence of breed on the formation of the saturated fatty acid composition of muscle tissue.

Component (g/100 g of Fat)	Breed			SEM	*p*-Value
	Limousin	Polish Holstein-Friesian	PHF × Limousin		
C12:0	0.06	0.09	0.05	0.011	0.991
C14:0	2.26 ^AB^	1.45 ^AC^	1.92 ^BC^	0.132	0.004
C16:0	25.57 ^Ab^	27.47 ^AC^	24.22 ^bC^	0.317	0.025
C18:0	18.40 ^aB^	19.26 ^aC^	16.87 ^BC^	0.153	0.000
SFA	48.30 ^aB^	49.74 ^aC^	43.99 ^BC^	0.752	0.000

aa, AA, etc.: Means in the same row marked with the same letters differ significantly at: small letters, *p* ≤ 0.05; capitals, *p* ≤ 0.01. SEM, standard error of LSM. SFA, saturated fatty acid.

**Table 5:** The influence of breed on MUFA and PUFA levels in muscle tissue.

Component (g/100 g of Fat)	Breed			SEM	*p*-Value
	Limousin	Polish Holstein-Friesian	PHF × Limousin		
C18:1 trans-11 (TVA)	1.13 ^ab^	0.83 ^aC^	1.31 ^bC^	0.071	0.048
C18:2 n-6 (LA)	8.70 ^Ab^	6.24 ^Ac^	7.97 ^bc^	1.041	0.021
C18:2 cis-9, trans-11 (CLA)	3.26 ^Ab^	2.59 ^AC^	3.59 ^bC^	0.147	0.033
C18:3 n-3 (LNA)	0.59 ^ab^	0.49 ^ac^	0.71 ^bc^	0.087	0.042
C20:5 n-3 (EPA)	0.58 ^a^	0.42 ^ab^	0.65 ^b^	0.064	0.049
C22:6 n-3 (DHA)	0.14 ^A^	0.07 ^AB^	0.16 ^B^	0.045	0.714
n-6 PUFA	11.01 ^A^	8.54 ^AB^	10.44 ^B^	0.121	0.036
n-3 PUFA	2.42 ^Ab^	2.06 ^AC^	2.81 ^bC^	0.175	0.006

aa, AA, etc.: Means in the same row marked with the same letters differ significantly at: small letters, *p* ≤ 0.05; capitals, *p* ≤ 0.01. SEM, standard error of LSM; PUFA, polyunsaturated fatty acid; MUFA, monounsaturated fatty acid.

**Table 6:** The influence of breed on bioactive protein fraction component levels in muscle tissue.

Component (mg/100 g)	Breed			SEM	*p*-Value
	Limousin	Polish Holstein-Friesian	PHF × Limousin		
Anserine	83.64 ^Ab^	61.22 ^AC^	81.46 ^bC^	1.170	0.000
Carnosine	462.48 ^AB^	387.3 ^AC^	492.36 ^BC^	8.226	0.000
Taurine	42.13 ^AB^	34.28 ^AC^	48.99 ^BC^	0.551	0.005
Coenzyme Q10	2.35 ^Ab^	1.87 ^AC^	2.67 ^bC^	0.125	0.044
Creatinine	5.48 ^AB^	4.12 ^AC^	6.36 ^BC^	0.054	0.037
Creatine	448.22 ^AB^	396.96 ^AC^	481.25 ^BC^	7.012	0.000

aa, AA, etc.: Means in the same row marked with the same letters differ significantly at: small letters, *p* ≤ 0.05; capitals, *p* ≤ 0.01. SEM, standard error of LSM.

**Table 7:** The influence of breed on vitamins soluble in fat in muscle tissue.

Component (μg/g)	Breed			SEM	*p*-Value
	Limousin	Polish Holstein-Friesian	PHF × Limousin		
β-carotene	0.29 ^AB^	0.20 ^AC^	0.45 ^BC^	0.003	0.000
α-retinol	0.75 ^AB^	0.66 ^AC^	0.92 ^BC^	0.008	0.018
α-tocopherol	3.14 ^AB^	1.61 ^AC^	4.76 ^BC^	0.098	0.024

aa, AA, etc.: Means in the same row marked with the same letters differ significantly at: capitals, *p* ≤ 0.01. SEM, standard error of LSM.
